# Thermodynamic Stability and Structural Insights for CH_3_NH_3_Pb_1−*x*_Si_*x*_I_3_, CH_3_NH_3_Pb_1−*x*_Ge_*x*_I_3_, and CH_3_NH_3_Pb_1−*x*_Sn_*x*_I_3_ Hybrid Perovskite Alloys: A Statistical Approach from First Principles Calculations

**DOI:** 10.1038/s41598-019-47192-7

**Published:** 2019-07-30

**Authors:** Diego Guedes-Sobrinho, Ivan Guilhon, Marcelo Marques, Lara K. Teles

**Affiliations:** Grupo de Materiais Semicondutores e Nanotecnologia, Instituto Tecnológico de Aeronáutica, DCTA, 12228-900 São José dos Campos, Brazil

**Keywords:** Structural properties, Sustainability, Thermodynamics

## Abstract

The recent reaching of 20% of conversion efficiency by solar cells based on metal hybrid perovskites (MHP), e.g., the methylammonium (MA) lead iodide, CH_3_NH_3_PbI_3_ (MAPbI_3_), has excited the scientific community devoted to the photovoltaic materials. However, the toxicity of Pb is a hindrance for large scale commercial of MHP and motivates the search of another congener eco-friendly metal. Here, we employed first-principles calculations via density functional theory combined with the generalized quasichemical approximation to investigate the structural, thermodynamic, and ordering properties of MAPb_1−*x*_Si_*x*_I_3_, MAPb_1−*x*_Ge_*x*_I_3_, and MAPb_1−*x*_Sn_*x*_I_3_ alloys as pseudo-cubic structures. The inclusion of a smaller second metal, as Si and Ge, strongly affects the structural properties, reducing the cavity volume occupied by the organic cation and limitating the free orientation under high temperature effects. Unstable and metaestable phases are observed at room temperature for MAPb_1−*x*_Si_*x*_I_3_, whereas MAPb_1−*x*_Ge_*x*_I_3_ is energetically favored for Pb-rich in ordered phases even at very low temperatures. Conversely, the high miscibility of Pb and Sn into MAPb_1−*x*_Sn_*x*_I_3_ yields an alloy energetically favored as a pseudo-cubic random alloy with tunable properties at room temperature.

## Introduction

Justified by the imminent scarcity of energy sources based on conventional fossil fuels, the recent rise of metal halide perovskites (MHP defined by ABX_3_) as alternative of low cost photovoltaic material has excited the community centered around silicon, which has been considered the principal element in solar cells^1–5^. MHP based on the use of lead iodide (${{\rm{PbI}}}_{3}^{-}$) and methylammonium (${{\rm{CH}}}_{3}{{\rm{NH}}}_{3}^{+}={{\rm{MA}}}^{+}$), i.e., MAPbI_3_^6–8^, reached remarkable 20%^9^ of efficiency in lighting conversion devices, which has put it as background for improvements of its photovoltaic performance^10–14^. However, a deeper comprehension of the chemical and structural properties correlated with the optical efficiency is needed. Additionally, for a large scale commercialization of solar cells based on MHP, combining thermodynamic stability and high photovoltaic performance is the key point for the viability of those devices^15–17^.

Experiments have revealed the MAPbI_3_ stability in different structural motifs into a relative short range of temperatures. For example, below 163 K the orthorhombic (*Amm*2 space group, $a=8.84$, $b=12.58$, $c=8.55$) is found^18^, between 163–328 K the structure becomes tetragonal (*I*4/*mcm* space group, $a=8.87$, $b=12.67$), and above 328 K^19^ MAPbI_3_ has been suggested as pseudo-cubic (*P*4*mm* space group, $a=6.31$)^20^. Additionally, the thermodynamic stability of MHP has been investigated aiming their obstacles against the inclement weather, such as UV, moisture, heat, and oxygen, which is crucial for MHP durability of photovoltaic cells^21–24^. Experiments of differential thermal analysis has indicated the decomposition of MAPbI_3_ tetragonal phase in CH_3_NH_3_PbI_3_(*s*) → PbI_2_(*s*) + CH_3_NH_2_(*g*) + HI(*g*), in order that for temperatures from 403 K the perovskite gradually starts to be decomposed^25^. It is reported through X-ray diffraction that even after the MAPbI_3_ systems be submitted under temperature of 443 K the sample keeps as 69% of MAPbI_3_ and 31% of PbI_2_^25^. For MASnI_3_, for instance, X-ray diffraction experiments revealed the presence of tetragonal structure at 423 K, and at room temperature by considering an MASn_*x*_I_3_ for $0.9\le x\le 1.4$ as relative quantities between MA:Sn^2+^ (in 1:*x*) used throughout the synthesis process, the perovskite adopts a pseudo-cubic structure for some *x* values^26^. However, the thermal decomposition starts only at 473 K, which is a higher than for MAPbI_3_.

From the last years the mixtures (alloys) MAPbI_3_-based perovskites has provided a new perspective to stabilize and tune MHP properties from their composition through several different ways, such as: (*i*) changing the MA^+^ organic cation by another keeping the charge balance^27–29^; (*ii*) replacing Pb^2+^ atoms by another cation, e.g., Sn^2+^ or Ge^2+^^30–33^; or (*iii*) varying the halogen^34–36^. This approach brought a tremendous progress in the development of MAPbI_3_-based for photovoltaic devices, especially for the MAPb_1−*x*_B_*x*_I_3_ alloys, from which the toxicity of Pb can be suppressed through the use of another congener eco-friendly metal (e.g. B = Sn or Ge)^37,38^. Based on that, those MHP alloys open an enhancement field for the photovoltaic performance by chemical control of the thermodynamic stability and optical properties^20,39–41^.

Even though MASnI_3_ has been investigated as an alternative for lead-free perovskite, its low power conversion efficiency^31^ and low oxidation resistence^30^ are some motivatory hindrances to workaround through the use of alloys. For instance, the MAPb_*x*_Sn_1−*x*_I_3_ stable alloy was recently investigated by Hao *et al*.^31^, who showed experimentally the control of the band gap of the MAPbI_3_ (1.55 eV) for compositions towards MASnI_3_ pure (1.30 eV), as the lower band gaps in 1.17 eV and 1.24 eV for MAPb_0.5_Sn_0.5_I_3_ and MAPb_0.75_Sn_0.25_I_3_. Furthermore, the study revealed that the MAPb_0.5_Sn_0.5_I_3_ alloy adopts a pseudo-cubic structure, while in so far as the content of Pb increases the structure adopts a tetragonal configuration, i.e., gradually reaching the stable phase of MAPbI_3_ at room temperature. In others studies focused on the optical and eletrochemical properties^30,42^, it was found an increase for the incident photon wavelength for MAPb_0.5_Sn_0.5_I_3_, which was red-shifted to 1060 nm, corresponding to the 260 nm displacement with respect to the MAPbI_3_ pure. Beyond that, since a large band gap of 1.90 eV for MAGeI_3_ has been found^43^, the MAPb_*x*_Ge_1−*x*_I_3_ alloy as tetragonal structure has also been investigated through theoretical calculations^44^. The alloys presented narrower band gaps than their pure perovskites counterparts, so that MAGe_0.75_Pb_0.25_I_3_ composition has presented the highest theoretical efficiency of about 24%. However, this study is restricted to few configurations and a deeper understanding of the structural stability is still needed.

As first attempt to determine the stability of a hibrid perovskite from a specific composition, the Goldschmidt’s tolerance factor (*t*) is a geometric parameter initially used to predict the ability to form a 3D perovskite^45^, which empirically lie into $0.80 < t < 1.1$ range^7,46^. The *t* is part of an empirical relation given by $({R}_{{\rm{A}}}+{R}_{{\rm{X}}})=t\sqrt{2}({R}_{{\rm{B}}}+{R}_{{\rm{X}}})$, where *R*_A_ is the effective radii of organic cation, *R*_B_ the radii for bivalent metal cation, *R*_X_ for halide anion. However, the Goldschmidt’s tolerance factor is limited to predict the perovskite alloys formation, since that parameters as the miscibility between the different metals involved within the crystal, i.e., concerning the octahedral inner sites occupied by Pb or a second metal B, as well as temperature relative to the thermodynamic favoring associated to the alloy stability, are crucial features for the comprehension of their electronic and atomic properties in dependence with the composition^47^. Furthermore, the Pb/B ratio for the metal size creates crystalline distortions (combined with the different magnitude for the spin-orbit contributions) which gives important insights for electronic characterization of those systems^48–50^. As such, a theoretical study for perovskite alloys needs a proper statistical approach relative to the configurational sampling constituting the statistical ensemble, which is required to calculate the average of thermodynamic and structural properties.

Here, we have performed first-principle calculations based on the Density Functional Theory (DFT) to investigate possible perovskite MAPbI_3_-based alloys. The generalized quasi-chemical approximation (GQCA) was used as statistical method, from which thermodynamic properties and averages of the structural parameters can be calculated for a wide chemical range at arbitrary temperatures. Thus, an improved picture on the perovskite alloys, since Si, Ge, and Sn metals present different relative atomic size with respect Pb, were studied in a pseudo-cubic MAPbI_3_ structure, considering their local impact on the structure for different direction within the crystal.

## Cluster Expansion and Thermodynamic Treatment

The structural and thermodynamic behaviour of the perovskite alloys were investigated through a rigorous and systematic statistical description based on the GQCA^51^. In the GQCA, the alloy (mixture) is described as an ensemble of clusters (herein our supercell), statistically and energetically independent of the surrounding atomic configuration. It has been demonstrated that this model successfully describes the physical properties of several 2D and 3D alloys, as well as to 2D sheets^52–55^. Furthermore, the GQCA method also has been employed in the thermodynamic analysis of perovskite alloys of MAPb(I_1−*x*_Br_*x*_)_3_^56^, however, the method was still not employed for perovsksite alloys from the metal perspective.

**Figure 1:**
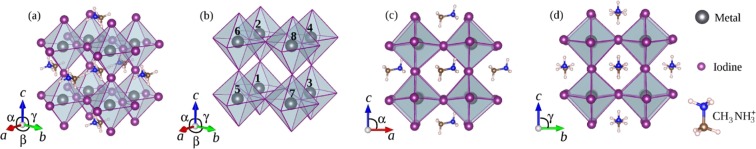
(**a**) Representation of the MAPb_1−*x*_B_*x*_I_3_ cubic supercell for all the perovskites and alloys based on metals B = Sn, Ge, and Si. (**b**) 8 sites in the ${{\rm{MI}}}_{3}^{-}$ octahedrals numbered to replacement of the metals and formation of the perovskite alloys. (**c**) Lateral disposition of the organic cations from the perspective of the *a* and *c* directions. (**d**) Perovskite with organic cations from the perspective of the *b* and *c* directions.

Within the GQCA size and shape of the clusters play an important role, wherein the supercell model has two advantages: (*i*) it has a reasonable size for taking into account the local correlation; and (*ii*) it has an exact counting scheme for the configurational entropy, since no two clusters share the same alloying atom. Based on that, the Fig. 1(a) shows a representation of a MHP as a cubic structure (symmetry group *O*_*h*_) with the CH_3_${{\rm{NH}}}_{3}^{+}$ cations balancing the ${{\rm{MI}}}_{3}^{-}$ anions charges of the octahedrals. We used a supercell with 2 × 2 × 2 expansion of a cubic perovskite by starting from the MAPbI_3_ system, from which the alloys are made by replacing the 8 octahedral central sites by Sn, Ge, and Si, named by the letter B in the general case, to build the CH_3_NH_3_Pb_1−*x*_Sn_*x*_I_3_, CH_3_NH_3_Pb_1−*x*_Ge_*x*_I_3_, and CH_3_NH_3_Pb_1−*x*_Si_*x*_I_3_ systems, respectively.

Regarding the 8 sites involving the replacement of 2 metal species, as shown in Fig. 1(b), the total number of possible atomic configurations is given by 2^*n*^, where *n* is the number of sites labeled by 12345678, i.e., resulting on 2^8^ = 256 possible configurations for each alloy. However, the 256 atomic configurations can be organized in $J=22$ symmetry equivalent classes by considering all the *O*_*h*_ space group operations. The Table 1 describes the 22 classes with respect the replacement of the octahedral sites, wherein Pb atoms are labeled by A and the Sn, Ge, and Si atoms by B.

**Table 1:** The 22 different cluster classes of MHP supercells with 8 sites in the ${{\rm{MI}}}_{3}^{-}$ octahedrals to study perovskite alloys with their *n*_*j*_ B atoms (Sn, Ge, and Si).

*j*	*n*_*j*_	Configuration 12345678	*g*_*j*_	*j*	*n*_*j*_	Configuration 12345678	*g*_*j*_
1	0	AAAAAAAA	1	12	4	AAABBBBA	24
2	1	AAAAAAAB	8	13	4	AABBBBAA	6
3	2	AAAAAABB	12	14	4	ABBABAAB	2
4	2	AAAAABBA	12	15	5	AAABBBBB	24
5	2	AAABBAAA	4	16	5	AABBBBAB	24
6	3	AAAAABBB	24	17	5	ABBABABB	8
7	3	AAABABBA	8	18	6	AABBBBBB	12
8	3	AAABBAAB	24	19	6	ABBABBBB	12
9	4	AAAABBBB	6	20	6	ABBBBBBA	4
10	4	AAABABBB	8	21	7	ABBBBBBB	8
11	4	AAABBABB	24	22	8	BBBBBBBB	1

The sequence 12345678 labeling the sites in the cluster can be found in Fig. 1(b), where A is Pb and B are the Sn, Ge, and Si atoms to each alloy, where *g*_*j*_ is the degeneracy factor.

**Figure 2:**
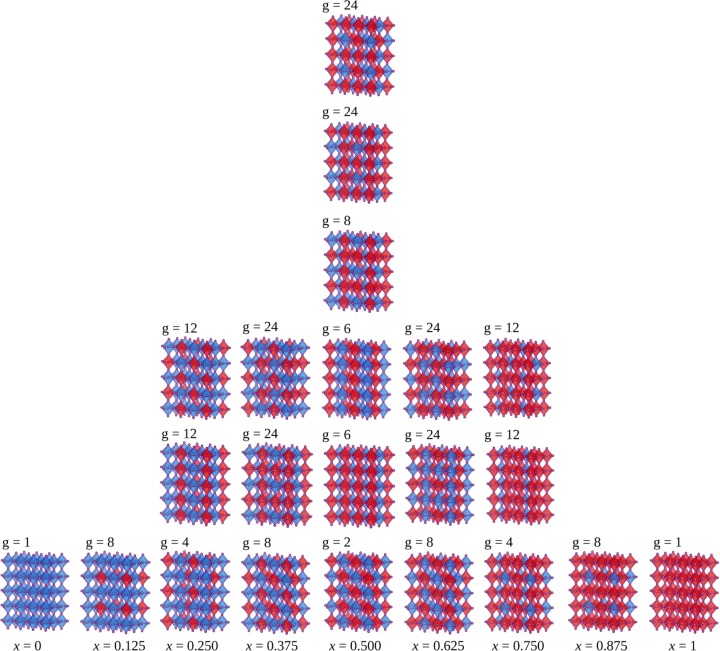
Representations of the MAPb_1−*x*_B_*x*_I_3_ (with B = Si, Ge, and Si) isomers for each class *J* for the *x* = 0, 0.125, 0.250, 0.375, 0.500, 0.625, 0.750, 0.875, 1 compositions. Above each structure, the degeneracy *g* as used into GQCA method is indicated.

The Fig. 2 shows a representation of the relative positions of the octhedral occupied by Pb (blue) and B (red) of the 22 classes, as well as their respective compositions *x* and degeneracies *g*_*j*_. Thereby, to describe our statistical ensemble for the perovskite alloys, we considered the set of 9 compositions, as *x* = 0, 0.125, 0.250, 0.375, 0.500, 0.625, 0.750, 0.875, 1, which were defined by the quantities of both metals involved in the alloy formation. Thus, for a given *N* as the total number of metals involved (or as the total number of sites occupied aforementioned), $x=\frac{{n}_{j}}{n}$ with *n*_*j*_ as the number of Sn, Ge, and Si atoms, and $n-{n}_{j}$ the number of Pb atoms in the cluster *j*. Thus, the excess energy of each of those *j* configurations among the 22 possibilities with internal mixing energy Δ${\varepsilon }_{j}$ can be defined by

1 $${\rm{\Delta }}{\varepsilon }_{j}={E}_{j}-(1-x){E}_{{\rm{MAPbI3}}}-x{E}_{{\rm{MABI3}}},$$

where, *E*_*j*_, ${E}_{{{\rm{MAPbI}}}_{3}}$, and ${E}_{{{\rm{MABI}}}_{3}}$ are the total energies of the cluster configuration *j*, the cluster of MAPbI_3_, and the cluster of MABI_3_ with B = Pb, Sn, Ge, and Si pure perovskites. As such, the internal energy is calculated by ${\rm{\Delta }}U(x,T)={\sum }_{j=1}^{j}\,{x}_{j}(x,T){\rm{\Delta }}{\varepsilon }_{j}$, where *x*_*j*_ is the probability distribution for the occurence of a cluster with configuration *j*. As described elsewhere^51,52,54,55^, the occurence probability *x*_*j*_ of equivalence class *j* can be estimated by the constrained minimization of the Helmholtz free energy, i.e., ${\rm{\Delta }}F(x,T)={\rm{\Delta }}U(x,T)-T{\rm{\Delta }}S(x,T)$, through the GQCA, by considering the probability normalization ${\sum }_{j=1}^{J}\,{x}_{j}(x,T)=1$ and average of composition *x* as calculated by ${\sum }_{j=1}^{J}\,{n}_{j}{x}_{j}(x,T)=nx$^51,52,57^. Thereby, the ${x}_{j}(x,T)$ distribution is given by

2 $${x}_{j}=\frac{{g}_{j}{\eta }^{{n}_{j}}{{\rm{e}}}^{-\beta {\rm{\Delta }}{\varepsilon }_{j}}}{{\sum }_{j=1}^{J}\,{g}_{j}{\eta }^{{n}_{j}}{{\rm{e}}}^{-\beta {\rm{\Delta }}{\varepsilon }_{j}}},$$

where $\beta ={({k}_{{\bf{B}}}T)}^{-1}$, and $\eta$ is an adimensional parameter obtained by the average composition constrain, and *g*_*j*_ is the degeneracy defined to each *j* as described in Table 1. The set of probabilities *x*_*j*_ permits to calculate any arbitrary property $p(x,T)$ for the alloy by

3 $$p(x,T)=\sum _{j=1}^{J}\,{x}_{j}(x,T){p}_{j},$$

where *p*_*j*_ is the local property of each cluster class *j*.

The mixing entropy in Δ$S(x,T)$ equation is calculated as

4 $$\begin{array}{rcl}{\rm{\Delta }}S(x,T) & = & -\,N{k}_{{\bf{B}}}[x\,\mathrm{ln}\,x+(1-x)\,\mathrm{ln}(1-x)]\\  &  & -\,M{k}_{{\bf{B}}}{D}_{KL}({x}_{j}\parallel {x}_{j}^{0}),\\ {\rm{where}}\,{D}_{KL}({x}_{j}\parallel {x}_{j}^{0}) & = & \sum _{j=1}^{J}\,{x}_{j}\,\mathrm{ln}(\frac{{x}_{j}}{{x}_{j}^{0}}),\end{array}$$

wherein *k*_**B**_ is the Boltzmann constant and *M* is the total of clusters. ${D}_{KL}({x}_{j}\parallel {x}_{j}^{0})$ is the Kullback-Leibler (KL) divergence as relative entropy measure, which evaluates the similarity (or dissimilarity) between the probability calculated through GQCA (*x*_*j*_) and the probability of the clusters *j* in an ideal solid solution (${x}_{j}^{0}$). The function ${x}_{j}^{0}$ as the random cluster probability distribution for the cluster *j* is calculated by ${x}_{j}^{0}={g}_{j}{x}^{{n}_{j}}{(1-x)}^{n-nj}$, by setting a reference from which ${D}_{KL}({x}_{j}\parallel {x}_{j}^{0})$ establishes the deviation of the mixing entropy and the one from the ideal system. Even though previous studies have reported the rotational activity for the methylamonium cations under high finite temperature effects^58–61^, which correlates with the typical range of synthesis temperature of MHP (300–400 K)^18,34,58,62^, intermittent rotational entropic contributions of the organic cations are not considered in our thermodynamic approach. Furhermore, the Δ${\varepsilon }_{j}$ values are predominantly determined by the octahedral configurations with sites occupied by Pb or B for the clusters *j*, as well as the spin-orbit coupling interation used in our calculations which comes only from the metals^63–65^. As such, we set all the cations oriented for the same direction as represented in Fig. 1(c,d), so that the relative directions in *a*, *b*, and *c* were defined as references for the structural analysis.

## Results and Discussion

We discuss the structural parameters, such as lattice parameters (on the orthogonal directions *a*, *b*, and *c*), local M-I distances (*d*^M–I^), angles I–M–I, between the lattice constants (*α*, *β*, and *γ*), and the volume (Å^3^) of the unit cell for the MAPb_1−*x*_B_*x*_I_3_ perovskite alloys as a function of the composition and temperature. By taking the Pb atom as reference, the atomic sizes decrease rising in the IV group of the periodic table, as B = Sn, Ge, and Si which are, respectively, 4.08, 17.01, and 32.65% smaller with respect to the Pb atom^66^. These differences in the atomic sizes of the metals correlated with the organic cation occupying the different cavity sizes made by the octahedrals, taking the relative orientations on the *a*, *b*, and *c* directions (as represented in Fig. 1(c,d)), permit a detailed atomistic comprehension for the pure and alloys perovskites in different compositions. Furthermore, a thermodynamic characterization is provided through the mixing internal energy (Δ*U*), mixing entropy (Δ*S*), excess of free energy (Δ*F*), as well as the construction of the $T-x$ phase diagram of the perovskite alloys.

### Structural Parameters of the Pure Perovskites and Their Alloys

#### Pure perovskites

The structural parameters for the MASiI_3_, MAGeI_3_, MASnI_3_, and MAPbI_3_ perovskites are shown in Table 2. All the structures adopt a pseudo-cubic structure ($P4mm$), in order the lattice constant values correlates with the atomic sizes of the metals into pseudo-cubic structures, i.e., *a*, *b*, and *c* follow MASiI_3_ < MAGeI_3_ < MASnI_3_ < MAPbI_3_. Our results are in good agreement with experimental reports, for MAPbI_3_^67^ our calculated lattice parameters deviate in $a=0.47 \%$, $b=-\,0.16 \%$, and $c=1.26 \%$, while for MASnI_3_^31^ in $a=0.96 \%$, $b=-\,0.48 \%$, and $c=0.64 \%$. For MAGeI_3_^68^, while our results are $a=1.22 \%$, $b=-\,1.88 \%$, and $c=0.24 \%$ with respect to the experimental values, MASiI_3_ still need accurated experimental structural parameters to compare.

**Table 2:** Lattice parameters, smallest and largest metal-halide distances (*d*^M–I^), M-I-M angles (${\varphi }^{{\rm{M}}-{\rm{I}}-{\rm{M}}}$) with respect to the *a*, *b*, and *c* directions, angles between the lattice constants (*α*, *β*, and *γ*), space group representation (SGR), and volume (*V*) of the unit cell for the MAPbI_3_, MASnI_3_, MAGeI_3_, and MASiI_3_ perovskites.

System	Space group	Lattice (Å)			Angles (°)			*d*^M–I^(Å)			*ϕ*^M–I–M^(°)			Volume (Å^3^)
	SGR	*a*	*b*	*c*	*α*	*β*	*γ*	*a*	*b*	*c*	*a*	*b*	*c*	*V*
MASiI_3_	*P*4*mm*	6.18	6.00	6.16	84	91	92	2.61	2.65	2.69	165	168	164	235.29
								3.62	3.38	3.53				
MAGeI_3_	*P*4*mm*	6.20	6.01	6.14	85	91	92	2.70	2.77	2.80	166	167	163	237.07
								3.56	3.28	3.42				
MASnI_3_	*P*4*mm*	6.30	6.21	6.32	88	90	90	2.91	3.12	3.05	173	169	170	258.25
								3.43	3.13	3.31				
MAPbI_3_	*P*4*mm*	6.35	6.31	6.40	90	90	90	3.02	3.17	3.18	173	167	167	265.79
								3.35	3.17	3.25				

We found that the smaller atomic size for Si and Ge when compared with Pb contributes to decrease the lattice constants in up to 4.91% (relative to the *b* direction) for both MASiI_3_ and MAGeI_3_ in comparison with MAPbI_3_. As consequence, their octahedrals are locally more distorted, as can be seen in Table 2 through the differences between the shortest and largest *d*^M–I^ values on all *a*, *b*, and *c* directions. We found that, in general, throughout the ${\rm{Si}} < {\rm{Ge}} < {\rm{Sn}} < {\rm{Pb}}$ sequence for the atomic size the shortest *d*^M–I^ distances increase while the largest *d*^M–I^ distances decrease, which is an effect of the competition of the metals into neighbor octahedrals by the I in the vertice between them. The angles values between the lattice constants (*α*, *β*, and *γ*) and the octahedral connection angles, i.e., ${\varphi }^{M-I-M}$, reveals that for MASnI_3_ and MAPbI_3_ the local distortions are similar, since their atomic sizes for Sn and Pb are similar. However, for MASiI_3_ and MAGeI_3_ the small metal occuping the octahedral sites promote higher deviations for the *α*, *β*, and *γ* angles with respect to the 90°, by leading also to the decreasing of the ${\varphi }^{M-I-M}$ on all directions also as a local distortion effect on the octahedrals.

Our unit cell volume results increasing as ${V}^{{\rm{MASiI3}}} < {V}^{{\rm{MAGeI3}}} < {V}^{{\rm{MASnI3}}} < {V}^{{\rm{MAPbI3}}}$ in correlation with the metal size, i.e., ${\rm{Si}} < {\rm{Ge}} < {\rm{Sn}} < {\rm{Pb}}$, suggest the same tendency relative to the cavity size where the organic cation is sited. For instance, the relative similarity between the MAPbI_3_ and MASnI_3_ pseudo-cubic structures also can be seen as a similar effect of the organic cation orientation on the *a*, *b*, and *c* lattice directions, yielding a low structural distortion on the pseudo-cubic motif and a low dependency of the structural parameters on *a*, *b*, and *c* directions with respect to the organic cation orientation. Consequently, the largest and shortest *d*^M–I^ values are similar on *b* for MAPbI_3_ (3.17 Å) and MASnI_3_ (3.13 Å) due to the CH_3_ and NH_3_ hydrogen, while on *a* and *c* the C-N bond axis its slope effects in the cavity are more pronounced on large and short *d*^M–I^ values. Conversely, as an effect of the small metal size and a smaller cavity volume, the stronger distortion observed for MASiI_3_ and MAGeI_3_ by comparing with MAPbI_3_ indicates a higher dependency relative to the organic cation orientation.

Therefore, we considered the momentary orientation of the organic cation to understand its effects on the ${{\rm{MI}}}_{3}^{-}$ inorganic octahedra. As such, Fig. 1(c,d) shows the MA^+^ C–N bond axis as momentarily oriented on *a*, giving the C-N bond axis sloped in the cavity on *b*, providing lowest energy configuration for the CH_3_NH_3_ group as reported by several atomistic simulation studies^56,69,70^. Thus, it is reasonable to expect that even though the high temperature effects promote the MA^+^ free reorientation in the cavity for MAPbI_3_, while the reorientation may be slightly limited in the MASiI_3_ and MAGeI_3_ pseudo-cubic structures.

#### Lattice parameters of the alloyed perovskites

The optimization of synthesis process of pure^4,18,34^ and alloy^30,31,41^ MHP at room temperature have widely been investigated, especially through self-assembling principles from the chemical precursors for the metal halides. As such, our statistic averages were calculated through GQCA at 300 K from the weighted contribution of each *j* configuration, providing the average of the structural parameters for the MAPb_1−*x*_B_*x*_I_3_ alloys as a function of the composition at room temperature.

**Figure 3:**
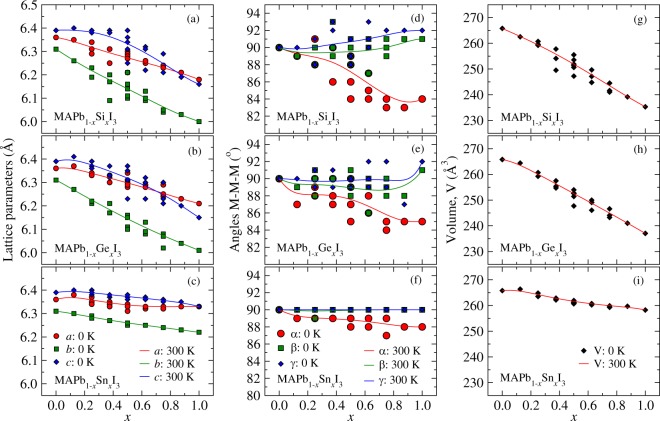
Lattice parameters (leftmost) in for the directions *a*, *b*, and *c*, angles (middle) between the lattice constants (*α*, *β*, and *γ*), and volume (rightmost) of the unit cell for the MAPb_1−*x*_Si_*x*_I_3_, MAPb_1−*x*_Ge_*x*_I_3_, and MAPb_1−*x*_Sn_*x*_I_3_ alloys. The symbols filled are the values for the configurations *j* and the solid lines are the average values within the GQCA calculated at 300.

We calculated the average lattice constants into the supercell on the *a*, *b*, and *c* directions, as well as the angles between them and the volume for the unit cell for each *j* cluster alloy (Fig. 3). Thus, the results connect the values for the MAPbI_3_ ($x=0$) and MABI_3_ ($x=1$), B = Si, Ge, and Sn. We found that the lattice parameters for the MAPb_1−*x*_Si_*x*_I_3_ (panel (a) in Fig. 3) alloy follow the Vegard’s law^71^ on the *a* and *b* directions, i.e., linearly decrease as a chemical specie with smaller atomic size is included into the bulk, while for the *c* direction it is observed a bowing. This result is due to the effects of the organic cation orientation taken as reference, wherein the C-N bond into the small cavity size yields different constraints on the lattice on the different directions. For example, on the plane made by *b* and *c* directions, on which the C-N bond of the CH_3_${{\rm{NH}}}_{3}^{+}$ is perpendicular, there is a deviation of the linearity with respect the composition as an effect of greater permissiveness of lattice distance adjustments with respect to the composition. Furthermore, as a consequence of the higher contraction of the lattice parameters as the Si atoms amount increases, we found a crossing over of the lattice parameters on the *a* and *c*, wherein the organic cation orientation yeilds lattice distances as $a < c$ and $a > c$ for the compositions $x < 0.875$ and $x > 0.875$, respectively.

For the MAPb_1−*x*_Ge_*x*_I_3_ alloy, the lattice parameter results were similar with the MAPb_1−*x*_Si_*x*_I_3_ (panel (b) in Fig. 3). We found that the Vegard’s law is followed for all the composition range for the *a* and *b* directions. The crossing over between *a* and *c* appears from $x > 0.750$, from which lattice parameters are $a > c$. Similarly, this result is also explained for the gradual contraction of the lattice parameters due to the small size of the Ge, as a consequence of the replacement of the Pb by Ge atoms, by yeilding a decreasing of the cavity size. As such, even though the C-N atoms of the MA^+^ are oriented perpendicular to the plane made by *b* and *c* orientations, the crossing over between *a* and *c* parameters for MAPb_1−*x*_Ge_*x*_I_3_ appears for lower quantities of Ge when compared with MAPb_1−*x*_Si_*x*_I_3_, which is a consequence of larger Ge size by comparing with Si.

For the MAPb_1−*x*_Sn_*x*_I_3_ lattice parameters shown into the panel (c) in Fig. 3, since the atomic sizes of the Pb and Sn atoms are similar there is no crossing over between *a* and *c* parameters, and the Vegard’s law is followed in all composition range connecting linearly the lattice parameter of the MAPbI_3_ and MASnI_3_ pure perovskites. As such, the linearity connecting the lattice parameters for the Pb-I-Pb, Pb-I-Sn, or Sn-I-Sn combinations are independent of the direction, suggesting that the pseudo-cubic structure for MAPb_1−*x*_Sn_*x*_I_3_ alloy is quite resistent with respect to the composition.

#### Lattice angles and volume of the alloyed perovskites

The panels (d), (e), and (f) in Fig. 3 show the lattice angles (*α*, *β*, and *γ*) for the MAPb_1−*x*_Si_*x*_I_3_, MAPb_1−*x*_Ge_*x*_I_3_, and MAPb_1−*x*_Sn_*x*_I_3_ alloys as a function of the composition. We found that *α* and *β* angles slightly increase between $x=0$ and $x=1$ for MAPb_1−*x*_Si_*x*_I_3_, lying into the interval 90°–92°. Conversely, *γ* decrease sharply with angle from 90° up to 84°, which is explained by the strong distortion on the pseudo-cubic structure due to the gradual replacement of Pb by Si atoms. Additionally, the volume of the unit cell for the MAPbI_3_ and MASiI_3_ pure perovskites are linearlly connected as function of the composition, with values lying between 265.79Å^3^ and 235.29Å^3^, which describes the constraction of the alloy by correlating with the Vegard’s law.

For MAPb_1−*x*_Ge_*x*_I_3_ alloys, we found that *α* and *β* are close to 90° between *x* = 0 and 0.875, while *γ* decrease sharply similarly with respect to the MAPb_1−*x*_Si_*x*_I_3_, that is between 90° up to 85°. This result shows the effects of the metals size differences, as well as the linear contraction for the volume of the unit cell between MAPbI_3_ and MAGeI_3_. This behaviour is also indicated for the *α* and *β* kept in 90° for the MAPb_1−*x*_Sn_*x*_I_3_ due to the similar size by comparing Pb and Sn, while *γ* lie into a short interval between 89°–90°.

#### M–I distances and M–I–M angles as local structural parameters

**Figure 4:**
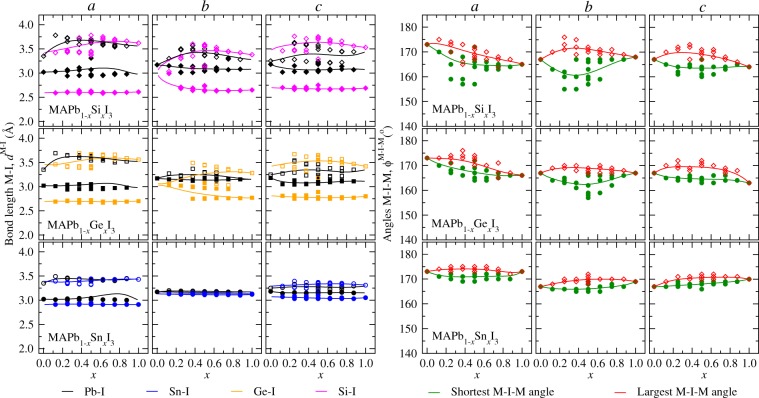
Shortest (filled symbols) and largest (empty symbols) M-I distances by M-I pair, i.e., *d*^M–I^ in (M = Si Ge, Sn, and Pb), and M-I-M angles each cluster *j*, as ${\varphi }^{{\rm{M}}-{\rm{I}}-{\rm{M}}}$ in (degrees), for the MAPb_1−*x*_Si_*x*_I_3_, MAPb_1−*x*_Ge_*x*_I_3_, and MAPb_1−*x*_Sn_*x*_I_3_ systems with respect to the directions *a*, *b*, and *c*, as a function of the alloy composition. The solid lines are the average values calculated within the GQCA calculated at 300.

To quantify the structural properties locally for the ${{\rm{MI}}}_{3}^{-}$ octahedrals with respect to the compositions, we calculated their shortest and largest M–I distances (*d*^M–I^) and M–I–M angles (${\varphi }^{{\rm{M}}-{\rm{I}}-{\rm{M}}}$) on the (*a*), (*b*), and (*c*) directions (Fig. 4). Once a supercell model was used in our calculations, the ${\varphi }^{{\rm{M}}-{\rm{I}}-{\rm{M}}}$ lie into different values between the shortest and largest M-I-M angles. Thus, the plotted ${\varphi }^{{\rm{M}}-{\rm{I}}-{\rm{M}}}$ values permit to describe the maximum amplitude of the local distortions relative to the compositions between $x=0$ and $x=1$. These averages calculated correspond to the equilibrium point relative to the equatorial anharmonic octahedral motion of the iodine atom in M–I–M^72^.

The shortest *d*^M–I^ values (Fig. 4 leftmost) in the alloys are determined by the Si-I, Ge-I, and Sn-I distances, which is an effect of the metal size differences with respect to the size of the Pb. One observes that for the *a* and *b* directions that for Pb-rich compositions the largest *d*^Pb–I^ values are higher than *d*^M–I^ values, wherein for few quantities of B the shortening of the B–I distance in an particular octahedral results in an elongation for the Pb–I distance relative to the neighbor octahedral. Thanks to these differences for the metal sizes into the clusters *j*, one observes an increasing of the amplitude for the shortest and largest *d*^M–I^ splitted from ${{\rm{MAPb}}}_{1-x}{{\rm{Sn}}}_{x}{{\rm{I}}}_{3}\to {{\rm{MAPb}}}_{1-x}{{\rm{Ge}}}_{x}{{\rm{I}}}_{3}\to {{\rm{MAPb}}}_{1-x}{{\rm{Si}}}_{x}{{\rm{I}}}_{3}$. This behaviour is explained by the local distortions on the octahedrals as the metal size differences are pronounced, also as an evidence of the organic cation influence on the inorganic lattice since the volume of the cavity decreases from $x=0$ to $x=1$. Furthermore, except for the *d*^M–I^ values for Pb-rich composition on *a* direction, our results show that the shortest and largest *d*^Pb–I^ values tends to keep as the those ones in the MAPbI_3_ pure perovskite, while the *d*^B–I^ values converge to the MABI_3_ pure values even for few quantities of B.

The ${\varphi }^{{\rm{M}}-{\rm{I}}-{\rm{M}}}$ values for each cluster *j* on all directions (Fig. 4 rightmost) highlight distortions into the pseudo-cubic alloys, herein stronger as the difference between the metals involved increases. For instance, for MAPb_1−*x*_Si_*x*_I_3_ the average ${\varphi }^{{\rm{M}}-{\rm{I}}-{\rm{M}}}$ values lie between 165°–175°, 160°–175°, and 165°–170° on the *a*, *b*, and *c* directions, respectively. One observes the effects of the strong local distortions induced by the presence of metals so different in size, e.g., Pb and Si, so that there is no linear correlation between the MAPbI_3_ and MASiI_3_ in the alloy formation. The MAPb_1−*x*_Ge_*x*_I_3_ alloy presents into softer distortion when compared with MAPb_1−*x*_Si_*x*_I_3_, as observed by the ${\varphi }^{{\rm{M}}-{\rm{I}}-{\rm{M}}}$ values into 165°–175°, 165°–170°, and 165°–170° intervals on the, respectively, *a*, *b*, and *c* directions. Moving to MAPb_1−*x*_Sn_*x*_I_3_, the ${\varphi }^{{\rm{M}}-{\rm{I}}-{\rm{M}}}$ values are similar from both MAPbI_3_ and MASnI_3_ pure perovskites, in order that small deviations appear between 170°–175° on the *a* direction and between 165°–170° on the both *b* and *c* directions.

With the results above discussed, we note the important role of the atomic size difference between the metals involved in the perovskite alloy formation. For MAPb_1−*x*_Sn_*x*_I_3_, as a case of similar size for the metals, the small local distortions into the octahedral and the linearity correlation between the MAPbI_3_ and MASnI_3_ pure perovskites show a preference in preserving the pseudo-cubic structure similar to the pure perovskites in the whole range of compositions. Conversely, the MAPb_1−*x*_Si_*x*_I_3_ and MAPb_1−*x*_Ge_*x*_I_3_ alloys are examples of large difference between the atomic size of the metals, we found that the composition is an additional variable with respect to the temperature to promotes strong distortions into the phase, reinforcing the necessity of a proper statistical analysis to correlates the thermodynamic stability with the structural motifs for the alloy.

### Thermodynamic Parameters and Ordering Preference

To predict the most favorable local arrangement of metal in the octahedral inner sites, i.e., the ${{\rm{PbI}}}_{3}^{-}$ and ${{\rm{BI}}}_{3}^{-}$ relative configuration, the alloy excess energies (Δ${\varepsilon }_{j}$) were calculated in order to determine the composition-dependent cluster probabilities (*x*_*j*_). Consequently, by knowing *x*_*j*_ as dependent of Δ${\varepsilon }_{j}$ and the degeneracies *g*_*j*_ for each *j*-configuration, we calculate the mixing free energy Δ$F(x,T)$ from the contributions of the interplay between the configurational entropy Δ$S(x,T)$ and the internal energy Δ$U(x,T)$ through the GQCA. As such, below we provide a thermodynamic discussion to enlighten the preferential ordering correlated to the stability of the MAPb_1−*x*_B_*x*_I_3_ perovskite alloys.

#### Alloy excess energies

**Figure 5:**
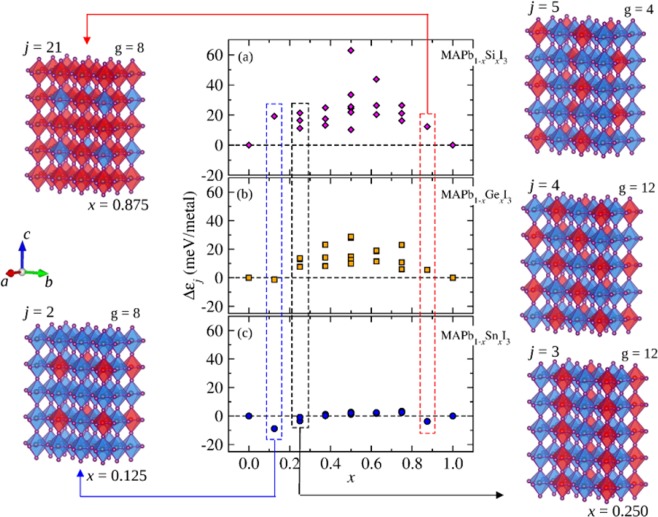
Excess energy (midle) in eV/metal for the each configuration *j* for the MAPb_1−*x*_Si_*x*_I_3_, MAPb_1−*x*_Ge_*x*_I_3_, and MAPb_1−*x*_Sn_*x*_I_3_ perovskite alloys. The MA were omitted for the representations of ${{\rm{PbI}}}_{3}^{-}$ (blue octahedrals) and ${{\rm{BI}}}_{3}^{-}$ (red octahedrals). The blue (leftmost) and red (rightmost) dashed boxes guide to the representation of the ordering for $x=0.125$ ($j=2$) and $x=0.875$ ($j=21$) with degeneracy $g=8$. Rightmost are the configurations $j=3$, $4$, and $5$ (black dashed box) for $x=0.250$ with degeneracies in $g=12$, $12$, and $4$, respectively.

The Fig. 5 provides a plot of the Δ${\varepsilon }_{j}$ values for the 22 considered cluster configurations as a function of the B, i.e., the metals Si, Ge, and Sn, as well as the arrangement representations (omitting the MA^+^ cations) of few configurations and their *g*_*j*_ values for some compositions *x*. For the MAPb_1−*x*_Ge_*x*_I_3_ and MAPb_1−*x*_Sn_*x*_I_3_ alloys, panels (b) and (c), respectively, the most energetically favorable configuration is for $x=0.125$, as represented by the arrangement correspondent to the Δ${\varepsilon }_{j}$ values indicated by the blue dashed box in Fig. 5.

The panel (a) shows all the positive Δ${\varepsilon }_{j}$ values for the MAPb_1−*x*_Si_*x*_I_3_ alloy, which means that at $T=0\,{\rm{K}}$ there is a high stability of the MAPbI_3_ and MASiI_3_ pure perovskites in detriment of the alloy. We found that all the pseudo-cubic configurations strongly distorted between $0 < x < 1$ lie into Δ${\varepsilon }_{j}$ values between 10 and 63 meV/metal, which is an evidence of the high strain yielded by the difference of the atomic size between Pb and Si. By comparing with the Ge alloy, in panel (b), an energetically favored cluster with Δ${\varepsilon }_{j}=-\,1.28\,{\rm{meV}}/{\rm{metal}}$ is observed at $x=0.125$, which correlates with a tendency to form a long-range ordered alloy depending on the temperature. However, all the distorted pseudo-cubic configurations for $0.125 < x < 1$ present Δ${\varepsilon }_{j}$ values between 7 and 30 meV/metal for MAPb_1−*x*_Ge_*x*_I_3_. This result suggests that for an MAPb_1−*x*_B_*x*_I_3_ (with B = Si, Ge, or Sn) perovskite alloy energetivally favorable two stability parameters are correlated: (*i*) the proportion (composition for the alloy) between the metals occupying the octahedral sites; and the (*ii*) magnitude of the atomic size difference between the metals involved.

As a consequence of small difference between the atomic size for Pb and Sn in the MAPb_1−*x*_Sn_*x*_I_3_, the Δ${\varepsilon }_{j}$ values lie in an interval of energies between −9 and 4 meV/metal. Thus, several configurations can be easily favorable when the entropy effects be considered. Therefore, as previously discussed for the structural parameters, such as the lattice parameters, *d*^M–I^, and *α*^M–I–M^ as a function of the *a*, *b*, *c* directions, this results suggest that the replacement of Pb by Sn yields only slight changing in the MAPb_1−*x*_Sn_*x*_I_3_ structure. Among all the configurations between $x=0$ and $x=1$ for the short range of Δ${\varepsilon }_{j}$ values for the MAPb_1−*x*_Sn_*x*_I_3_ alloy, additionally to the $x=0.125$ (−8.82 meV/metal) and $x=0.875$ (−3.77 meV/metal) compositions showed in Fig. 5, the three possible configurations at $x=0.250$ are represented by *j* = 3, 4, and 5, which present Δ${\varepsilon }_{j}$ in −3.43, −0.88, and −0.82 meV/metal respectively. We observe that the ordering *j* = 3 as represented in Fig. 5 is the most favored, in which the stability is reached by the stacking of the intercalated ${{\rm{PbI}}}_{3}^{-}$ and ${{\rm{SnI}}}_{3}^{-}$ octahedral rows.

#### Perovskite alloys free energies and ordering

**Figure 6:**
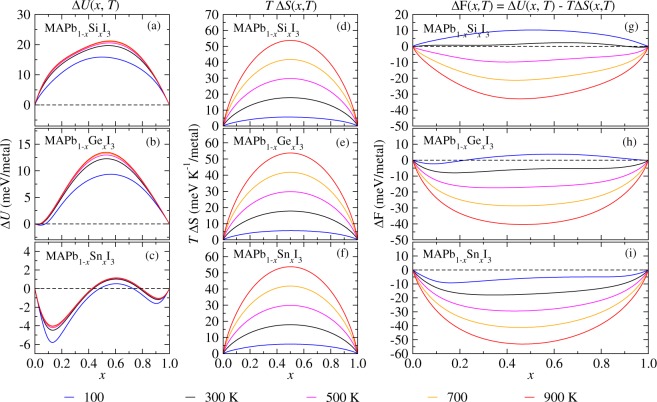
Thermodynamic parameters as a function of the alloy composition and temperature for MAPb_1−*x*_Si_*x*_I_3_, MAPb_1−*x*_Ge_*x*_I_3_, and MAPb_1−*x*_Sn_*x*_I_3_ calculated within the GQCA at 100, 300, 500, 700, and 900 K. Panels (a–c) are the averages of the internal energies in m/metal (Δ*U*); panels (d–f) are the averages of the entropy contribution as a function of the temperature in meVK^−1^/metal (*T*Δ*S*); and panels (g–i) are the Helmholtz free energy in m/metal (Δ*F*).

Here, we discuss the statistical contributions of the Δ${\varepsilon }_{j}$ values for the thermodynamic properties for the alloys under the temperature effects through the GQCA method. The variation in the energy of mixing (Δ*U*) and entropy of mixing (Δ*S*) used to calculate the Helmholtz free energies (Δ*F* in m/metal) for the MAPb_1−*x*_Si_*x*_I_3_, MAPb_1−*x*_Ge_*x*_I_3_, and MAPb_1−*x*_Sn_*x*_I_3_ alloys within the GQCA are shown in Fig. 6. In order to verify the entropy effects for the stabilities of the alloys, we analysed these parameters as a function of low and high temperatures, e.g., 100, 300, 500, 700, and 900 K.

One observes by the Δ*S* symmetrical curves with temperature around $x=0.500$, panels (d), (e), and (f), indicating that all the alloys follow an ideal entropy expression at high temperatures, i.e., $-\,N{k}_{{\bf{B}}}\,[x\,\mathrm{ln}\,x+(1-x)\,\mathrm{ln}(1-x)]$. The Δ*U* curves for MAPb_1−*x*_Si_*x*_I_3_ – panel (a) – present a positive parabolic behaviour due to the higher stability of the MAPbI_3_ and MASiI_3_ pure perovskites in comparison with the alloy. Thus, the profile of the Δ*U* and Δ*S* curves indicates that the alloy can be stabilized by entropic contributions, consequently by increasing the magnitude of disorder through the insertion of Si atoms, which promotes the contribution of several *j* configurations. The panel (g) shows a behaviour slightly asymmetric for the Δ*F* curve around $x=0.500$, so that for $T < 300\,{\rm{K}}$ we found Δ$F > 0$ as an evidence of the instability of the alloy at low temperatures. However, for $T > 300\,{\rm{K}}$ one observes that Δ$F < 0$ and the alloy starts to be stable, and for temperatures between $300\,{\rm{K}} < T < 500\,{\rm{K}}$ there are points throughout Δ*F* with same tangent, indicating the existence of a miscibility gap for an extensive range of temperatures.

For the MAPb_1−*x*_Ge_*x*_I_3_ alloy, we found that the Δ*F* – panel (h) – presents points with same tangent for $100\,{\rm{K}} < T < 500\,{\rm{K}}$, which is a range of lower temperatures for the miscibility gap than for MAPb_1−*x*_Si_*x*_I_3_. The Δ*F* reaches symmetrical curves for temperatures higher than 500 K, which the entropy effects start to be dominant over the small negative Δ*U* values, panel (b), for few Ge quantities. Conversely, with the increasing of the temperature, the disordering is reached with the weighted contributions of all ${{\rm{PbI}}}_{3}^{-}$ and ${{\rm{GeI}}}_{3}^{-}$ octahedrals configurations, from which the random configurations for $x=0.500$ compositions are the most favorable.

The Δ*U* curves profile for the MAPb_1−*x*_Sn_*x*_I_3_ alloy – panel (c) – show the effect of the favorable ordering for compositions with excess of both Pb and Sn metals, as *x* = 0.125, and 0.875. Firstly, this yields two regions for Δ$U < 0$ relative to the orderings as represented in Fig. 5, so that the alloy stabilizes when the ${{\rm{SnI}}}_{3}^{-}$ individual octahedrals are completely involved by ${{\rm{PbI}}}_{3}^{-}$ octahedrals, as well as for the opposite configuration, i.e., ${{\rm{PbI}}}_{3}^{-}$ individual octahedrals completely involved by ${{\rm{SnI}}}_{3}^{-}$. Secondly, the short range of excess energies for MAPb_1−*x*_Sn_*x*_I_3_ yields a short interval of Δ*U* variation as a function of the composition and temperature. Thus, for temperatures higher than 100 K the entropy effects are dominant, so that the shape of the Δ*F* curve becomes more symmetric in order that the contribution of all configurations increases with the temperature, consequently, increasing the disordering of ${{\rm{PbI}}}_{3}^{-}$ and ${{\rm{SnI}}}_{3}^{-}$ positions in the alloy. As such, it is expected to observe a miscibility gap in MAPb_1−*x*_Sn_*x*_I_3_ alloy with pseudo-cubic structure only for very low temperatures, since there is no effective variation of the structural environment when the Pb in the octahedral sites are replaced by Sn, which is a result of the almost similar atomic size between both metals.

**Figure 7:**
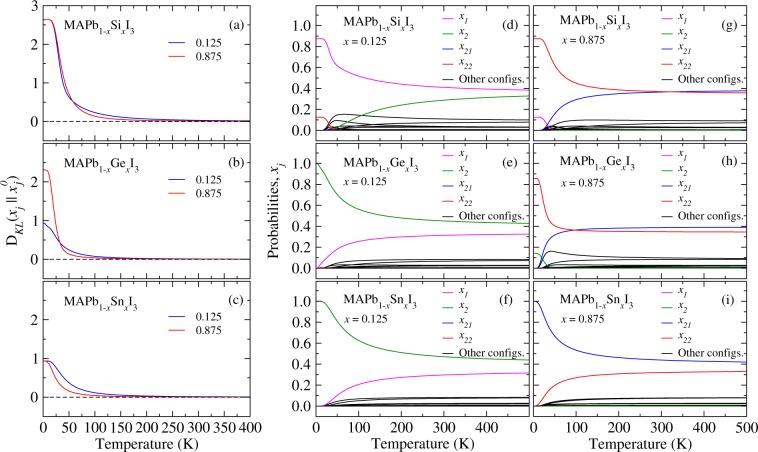
(Leftmost) Kullback-Leibler divergence – *D*_*KL*_(${x}_{j}\parallel {x}_{j}^{0}$) – for all the alloys between the ideal solid solution and GQCA probability distributions and probabilities *x*_*j*_ (rightmost) for the ordering $j=1$, $2$, $21$, and 22 as a function of temperature and compositions at *x* = 0.125 and 0.875.

To investigate the similarity between the GQCA probability ${x}_{j}(x,T)$ and a random alloy, relative to the random contribution of a particular *j* configuration in a range of temperatures, we present the KL divergence, namely, *D*_*KL*_(${x}_{j}\parallel {x}_{j}^{0}$), Fig. 7. The maximum divergence at low temperatures means that in a particular composition the configuration *j* relative to the *x*_*j*_ dominates over the others, and in so far as the temperature increases the divergence goes to zero, i.e., the system starts to behave as a random alloy. In Fig. 7(a–c) are plotted the *D*_*KL*_(${x}_{j}\parallel {x}_{j}^{0}$) for MAPb_1−*x*_Si_*x*_I_3_, MAPb_1−*x*_Ge_*x*_I_3_, and MAPb_1−*x*_Sn_*x*_I_3_ for the compositions at *x* = 0.125 and 0.875 as a function of the temperature. For MAPb_1−*x*_Si_*x*_I_3_ at very low temperatures one observes a tendency for phase segregation with the formation of MAPbI_3_ and MASiI_3_ pure perovskites, as observed through *x*_*j*_ plots in Fig. 7(d,g), wherein there is a predominancy of *x*_1_ and *x*_22_ configurations at compositions *x* = 0.125 and 0.875, respectively. For MAPb_1−*x*_Ge_*x*_I_3_, panel (b), clearly it is seen that at $x=0.125$ the divergence is smaller than for $x=0.875$ at very low temperatures, so that the ordering given by the ${x}_{2}=1$ configuration dominates at $x=0.125$ composition for the alloying at Pb-rich compositions (panel (e)), as well as yields a small contribution at $x=0.875$ together with the dominant *x*_22_ configuration (panel (h)). Conversely, for MAPb_1−*x*_Sn_*x*_I_3_ at $x=0.125$ and $x=0.875$ and at low temperature, panel (c), one observes the high miscibility between Pb and Sn as an effect of the similar metal size. Thereby, the *x*_*j*_ plots, panels (f) and (i), show the predominancy of the ${x}_{2}=1$ (${x}_{21}=1$) at $x=0.125$ ($x=0.875$) at 0 K, demonstrating the tendency of the system in organizing itself in energetically favored alloyed configurations even at very small temperatures. The observed ordering of atomic distribution, however, does not persist for temperatures above 150 K.

#### Phase diagram of the alloys

**Figure 8:**
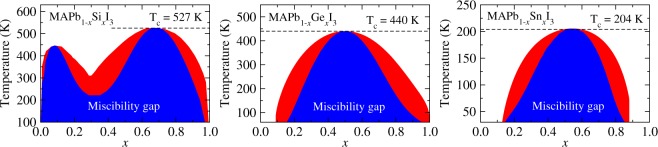
Predicted phase diagram of the MAPb_1−*x*_Si_*x*_I_3_, MAPb_1−*x*_Ge_*x*_I_3_, and MAPb_1−*x*_Sn_*x*_I_3_ alloys at pseudo-cubic structure. The blue and red regions are the miscibility gap (spinodal line) and metaestability (defined by the binodal line) regions, respectively, while the white region is the stable solid-solution with respect the temperature and compositions. The dashed line indicates the critical temperature (*T*_*c*_) for each alloy.

To identify regions of stability and metaestability as a function of the temperature and composition, we built the phase diagram for the alloys at the pseudo-cubic structure, as shown in Fig. 8. We observe for MAPb_1−*x*_Si_*x*_I_3_ (leftmost), MAPb_1−*x*_Ge_*x*_I_3_ (middle), and MAPb_1−*x*_Sn_*x*_I_3_ (rightmost) critical temperatures (*T*_*c*_) of 527, 440, and 204 K, respectively. Above *T*_*c*_ the solid solution are stable for any composition, whereas below them are evidenced the presence of miscibility gaps to each alloy defined by spinodal lines, given by ${x^{\prime} }_{1}$ and ${x^{\prime} }_{2}$ (blue regions), as well as binodal lines from the ${x}_{1}$ and ${x}_{2}$ points defining the $T-x$ metaestability regions.

For MAPb_1−*x*_Si_*x*_I_3_, due to the Δ*F* profile observed especially for its formation at room temperature (300 K), as shown in Fig. 6, its phase diagram presents unstable regions from Pb- to Si-rich compositions at low temperatures, yielding two miscibility gaps in dependence of the composition region. For instance, for Pb-rich compositions the first miscibility gap lies between ${x^{\prime} }_{1}=0.02$ and ${x^{\prime} }_{2}=0.19$, whereas the second one, relative to the Si-rich compositions, lies in the interval of ${x^{\prime} }_{1}=0.45$ up to ${x^{\prime} }_{2}=0.89$. One observes that the first miscibility gap reduces as the temperature increases up to 445 K, from which a solid solution is formed for Pb-rich compositions. However, only from ${T}_{c}=527\,{\rm{K}}$ at $x=0.68$ the solution solid is stable into all composition interval. Furthermore, from the end of the first miscibility gap up to the beginning of the second one, i.e., between the set of compositions into *x* = 0.19 − 0.45, the alloy present a metaestable phase resistant to the thermal fluctuations due to the valley yielded by the configurations *j* indicated within the dashed retangle in Fig. 5.

For the MAPb_1−*x*_Ge_*x*_I_3_ alloy, a stable solid solution is observed in all range of temperatures only for Pb-rich compositions between $0 < x < {x}_{1}$ for ${x}_{1}=0.20$, while at 300 K the miscibility gap appears between ${x^{\prime} }_{1}=0.31$ and ${x^{\prime} }_{2}=0.70$. At 400 the intervals for miscibility gap and metaestable phases are shorter than at room temperature. By comparing the MAPb_1−*x*_Si_*x*_I_3_ and MAPb_1−*x*_Ge_*x*_I_3_ alloys at compositions Si-, Ge-, and Pb-rich, one observes a behavior very different due to the effect of the Pb/Si and Pb/Ge metal size differences. Even though there is a stability of the MAPb_1−*x*_Ge_*x*_I_3_ alloy for Pb-rich into all temperatures, the symmetrical-like spinodal line at 300 K yields a stability for a range of Ge-rich compositions. Additionally, metaestable phases are observed into *x* = 0.20 − 0.31 and *x* = 0.70 − 0.81 intervals of compositions.

We found that the critical temperatures *T*_*c*_ for MAPb_1−*x*_Si_*x*_I_3_, MAPb_1−*x*_Ge_*x*_I_3_, and MAPb_1−*x*_Sn_*x*_I_3_, from which the solid solution at all compositions is stable, correlates with the atomic size difference for the metals involved. For example, the Fig. 8 shows also the phase diagram for the MAPb_1−*x*_Sn_*x*_I_3_, in which one observes the effects of small difference between the Pb and Sn atomic size from the high solubility of the metals into MASnI_3_ and MAPbI_3_, respectively. Since the critical temperature is ${T}_{c}=204\,{\rm{K}}$, at 300 K a stable solid solution is observed within all range of compositions, which is in agreement with Hao *et al*.^31^ experiments for the synthesis of MAPb_1−*x*_Sn_*x*_I_3_ who observed a high stability of the pseudo-cubic structure of the MAPb_0.5_Sn_0.5_I_3_ alloy, as well as in others compositions. Furthermore, we found a miscibility gap slightly symmetrical for MAPb_1−*x*_Sn_*x*_I_3_ appearing only at very low temperatures, since the local distortions into the structure are suppressed and the entropic effects are restricted to the configurations of the ${{\rm{PbI}}}_{3}^{-}$ and ${{\rm{SnI}}}_{3}^{-}$ octahedrals. Therefore, by taking as reference $T=443\,{\rm{K}}$ and $T=473\,{\rm{K}}$ as experimental temperatures in which the MAPbI_3_ and MASnI_3_ pure perovskites start to be decomposed^25,26^, our results show that there is a range of temperatures from ${T}_{c}=204\,{\rm{K}}$ in which the MAPb_1−*x*_Sn_*x*_I_3_ is stable as a random alloy before a possible thermal decomposition. Furthermore, for the others alloys, those results may be as a guide for future synthetic process for the MAPb_1−*x*_Si_*x*_I_3_ and MAPb_1−*x*_Ge_*x*_I_3_ alloys, from which it is expected the phase segregations for some range of compositions.

## Conclusions

In summary, we have performed first-principles calculations combined with a statistical approach based on cluster expansion to study the stability, effects of disorder, distortions, thermodynamic properties, and phase segregation of the pseudo-cubic phase of MAPb_1−*x*_B_*x*_I_3_ alloys for B = Si, Ge, and Sn.

Our results indicated that the metal atomic size plays an important role on the pseudo-cubic properties of the pure perovskites, e.g., as the similar local distortions for the MAPbI_3_ and MASnI_3_ octahedrals since their metals have almost the same atomic size. As such, the MAPb_1−*x*_Sn_*x*_I_3_ alloy presents lattice parameters in good agreement with the Vegard’s law for the whole range of compositions, wherein the alloy adopts a random ${{\rm{PbI}}}_{3}^{-}$ and ${{\rm{SnI}}}_{3}^{-}$ octahedral configurations. Conversely, MASiI_3_ and MAGeI_3_ in pseudo-cubic structure are strongly distorted as an effect of their second smaller metal in comparison with Pb, suggesting a higher limitation of the organic cation orientation on the lattice directions for the MAPb_1−*x*_Si_*x*_I_3_ and MAPb_1−*x*_Ge_*x*_I_3_ alloys, since the cavity volume is reduced. For those cases, the alloys follow the Vegard’s law for some particular lattice directions, whereas the others there is a pronounced bowing throughout the range of compositions.

The thermodynamic results revealed that the MAPb_1−*x*_Ge_*x*_I_3_ alloy is stable for Pb-rich compositions, i.e., between $0 < x < 0.20$ at 300 K, by presenting a preference for an ordered configuration in which one ${{\rm{GeI}}}_{3}^{-}$ octahedral is surrounded by ${{\rm{PbI}}}_{3}^{-}$ octahedrals. Conversely, MAPb_1−*x*_Si_*x*_I_3_ is not favored into very large range of compositions, and even though has presented an interval of compositions into which the alloy is metaestable (into *x* = 0.19 − 0.45), it indicated a high tendency for segregation phase in MAPbI_3_ and MASiI_3_ pure perovskites. Thus, the addition of small metal atoms yields strong local distortions on the octahedrals, resulting in very high critical temperatures for these alloys. As an exemple of miscibility, the MAPb_1−*x*_Sn_*x*_I_3_ alloy presented a critical temperature lower than the room temperature, at 204 K, which is very lower than the temperature of decomposition for the MAPbI_3_ and MASnI_3_ pure perovskites. Thus, the alloy is favored as a random alloy in all compositions, revealing that there is a safe range of temperatures in which the MAPb_1−*x*_Sn_*x*_I_3_ alloy properties can be tuned before the material be thermally decomposed.

Therefore, beyond the temperature as variable, the correlation between composition and atomic size, relative to the second metal in MAPbI_3_-based alloys, is a crucial element to promotes the phase stability. The thermodynamic characterization of these alloys for intermediate Pb-based compositions showed the importance of the planning relative to the experimental synthesis conditions, such as temperature and composition, aiming the structural motifs correlated with their performance into solar cells devices.

### Theoretical Approach and Computational Details

In this study, to calculate the total energy of the configurations of the alloy in all the range of compositions, we employed spin-polarized calculations based on DFT^73,74^ within the semilocal Perdew–Burke–Ernzerhof^75^ (PBE) formulation for the exchange-correlation energy functional. The projected augmented wave^76,77^ (PAW) method as implemented in the Vienna *ab initio* simulation package (VASP), version 5.4.1.^78,79^ was used to solve the Kohn–Sham (KS) equations, in which the scalar-relativistic approximation is considered to the core states, as well the spin-orbit coupling (SOC) interactions. However, SOC is an important relativistic phenomenon in Pb-based perovskites^63–65^, especially occurring within non-spherical atomic orbitals and affecting the directionality of the metal bonds^7^, so that we included SOC interactions also for the valence states in all our calculations.

For total energy calculations, we employed a plane-waves expansion with kinetic energy cutoff of 500, by integrating over the Brillouin zone calculated considering a Monkhorst-Pack **k**-mesh of 4 × 4 × 4. The total energy convergence to 1.0 × 10^−5^ eV with Gaussian broadening parameter of 50 for all calculations. Finally, the equilibrium of the Hellmann-Feynman forces on every atom were reached to forces smaller than 0.010 eV/Å.
